# Supplementary material for: Community involvement works where enforcement fails: conservation success through community-based management of Amazon river turtle nests
Source: PeerJ. 2018 Jun 1;6:e4856. doi: 10.7717/peerj.4856 (PMC5985759; doi:10.7717/peerj.4856)
Supplement: Figure S1 [file peerj-06-4856-s001.docx]

S1 Community management of nesting areas.

Team installing predator exclusion devices to protect yellow spotted river turtle nests. Photo credit: James Gibbs.

| 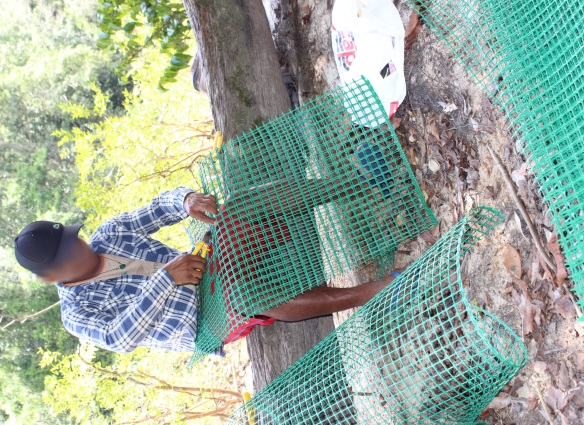 | 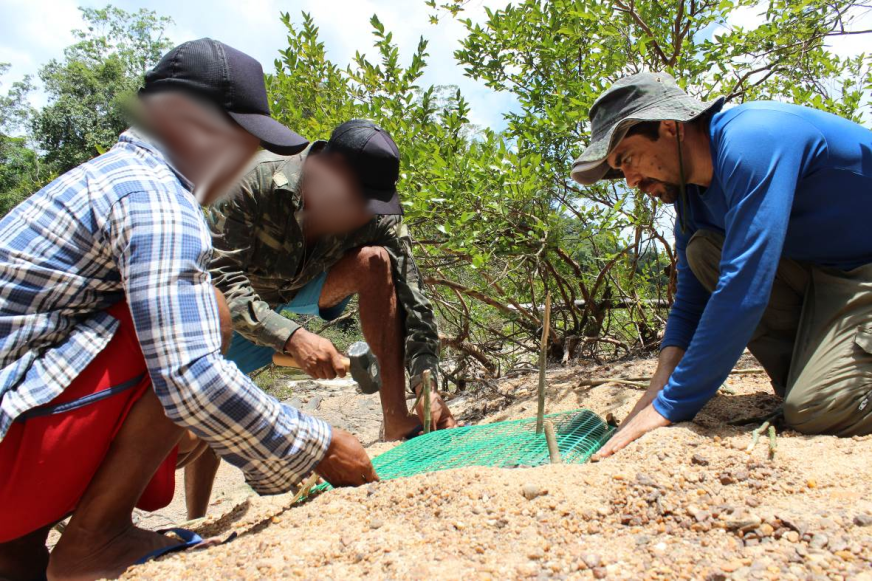 | |
| --- | --- | --- |
| 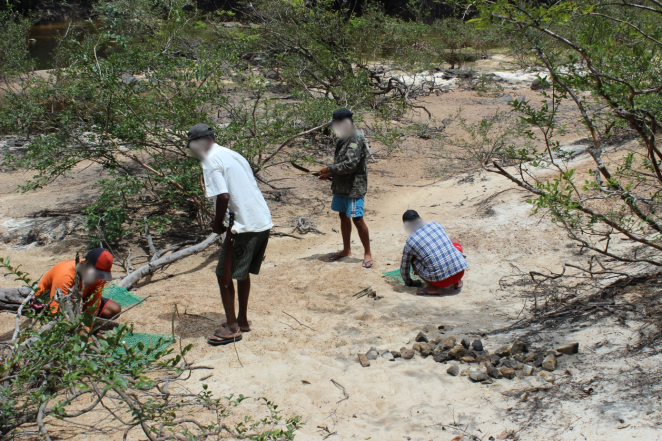 | | 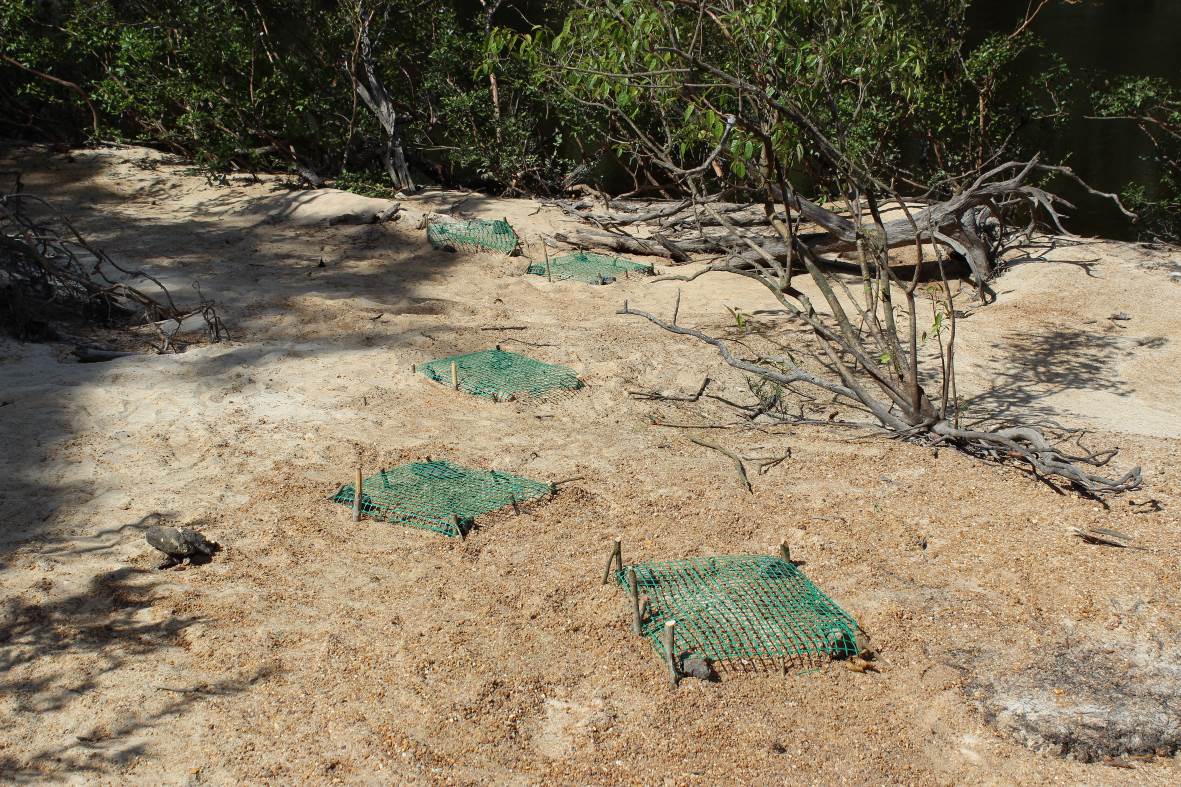 |
